# Monascin abrogates RANKL-mediated osteoclastogenesis in RAW264.7 cells via regulating MAPKs signaling pathways

**DOI:** 10.3389/fphar.2022.950122

**Published:** 2022-07-15

**Authors:** Yin Cheng, Haixia Liu, Jing Li, Yujie Ma, Changheng Song, Yuhan Wang, Pei Li, Yanjing Chen, Zhiguo Zhang

**Affiliations:** ^1^ Institute of Basic Theory, China Academy of Chinese Medical Sciences, Beijing, China; ^2^ Institute of Basic Medical Sciences Chinese Academy of Medical Sciences, School of Basic Medicine Peking Union Medical College, Center of Excellence in Tissue Engineering, Chinese Academy of Medical Sciences; Beijing Key Laboratory of New Drug Development and Clinical Trial of Stem Cell Therapy (BZ0381), Beijing, China

**Keywords:** monascin, osteoclast, RAW264.7, bone resorption, mapks, osteoporosis

## Abstract

Osteoclasts (OCs) are multinucleated cells that play a major role in osteolytic diseases such as osteoporosis. Monascin (Ms) is one of the active substances in the traditional Chinese medicine red yeast rice. Studies have found that red yeast rice can maintain bone health. In this study, the anti-osteoclastogenesis effects of Ms on RANKL-induced RAW264.7 cells were assessed, and the underlying mechanism was investigated. Ms exhibited inhibitory effects on OC differentiation and formation in a dose-dependent manner and suppressed the bone-resorbing activity of mature OCs. Ms blocked OCs-typical genes (c-Fos, NFATc1, CSTK, MMP-9, TRAP, ITG-β3, OSCAR and DC-STAMP). Furthermore, Ms treatment considerably inhibited the activation of MAPKs, JNK and p38. Taken together, Ms suppresses RANKL-induced osteoclastogenesis of RAW264.7 cells by restraining MAPKs signaling pathways and is a potential therapeutic option as a novel OC inhibitor to mitigate bone erosion.

## Introduction

The bones of human body provide surprisingly sophisticated support for the entire body as they protect the internal organs and are involved in metabolism (Karsenty and Ferron, 2012; Wang et al., 2021). As a mineralized connective tissue, bones are dynamically remodeled throughout life, consisting of three consecutive and cyclic phases: resorption, reversal and formation (Siddiqui and Partridge, 2016; Chen et al., 2018). Several bone diseases result from the disruption of the balance between osteoclasts (OCs) and osteoblasts. Excessive OC bone resorption results in osteoporosis and rheumatoid arthritis, characterized by low bone density, whereas the contrary may contribute to osteopetrosis, marked by high bone density (Jacome-Galarza et al., 2019; Blangy et al., 2020). Inhibiting OC formation and function is a viable strategy for treating low bone density diseases.

OCs are terminally differentiated macrophage multinucleated cells tightly attached to the bone surface, derived from myeloid mononuclear cells. Their differentiation depends on multiple factors (Asagiri and Takayanagi, 2007). Previous research reported the essential roles of the macrophage colony-stimulating factor (M-CSF) and the receptor activator of nuclear factor kappa-B ligand (RANKL) in OC differentiation, formation and function (Song et al., 2019; Zhao et al., 2019; Kitaura et al., 2020). M-CSF not only stimulates the proliferation and survival of the OC precursor cells but also upregulates RANK expression (Asagiri and Takayanagi, 2007). The RANKL-induced osteoclastogenesis is a sequential process comprising three stages: integration to its receptor RANK to recruit the adaptor molecule TNF receptor-associated factor 6 (TRAF6), resulting in mitogen-activated protein kinases (MAPKs) and nuclear factor-kappa B (NF-κB) activation, and promoting the expression of OCs-typical genes (Sun et al., 2021).

MAPKs signal pathways are indispensable in OC differentiation and bone resorption, including extracellular signal-regulated kinase (ERK), c-Jun N-terminal kinase (JNK), and p38 signaling (Lee et al., 2018). ERKs regulate the transcription factors c-Fos and nuclear factor of activated T-cells, cytoplasmic (NFATc) 1, which are involved in the differentiation, migration and function of OCs(He et al., 2011). JNK signaling is activated by RANKL through inflammatory cytokines such as TRAF6, mainly affecting the fusion and tartrate-resistant acid phosphatase (TRAP) activity of OC precursors (Chang et al., 2008; Ikeda et al., 2008). RANKL-RANK signaling induces p38 phosphorylation and NF-κB activation (Matsumoto et al., 2000), which directly influences the fusion factors such as dendrocyte-expressed seven-transmembrane protein (DC-STAMP) and ITG-β3, as well as function factors, including TRAP, cathepsin K(CTSK) and matrix metalloproteinase 9 (MMP-9) (Mansky et al., 2002; Herbert et al., 2015).

**FIGURE 1:**
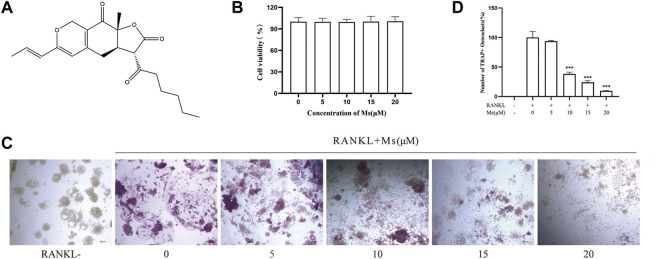
Inhibitory effect of Ms on RANKL-induced osteoclast formation *in vitro*. **(A)** Chemical structure of Ms. **(B)** Viability of RAW264.7 cells. RAW264.7 cells exposed to 5, 10, 15 and 20 μM Ms were assessed by CCK-8 kits after 72 h. **(C)** The osteoclast morphology after incubation with RANKL and Ms at 5, 10, 15 and 20 μM for 5 days. TRAP-positive cells (TRAP (+)) (nuclei≥3) were photographed by a light microscope, original magnification ×200 (Scale bar = 200 μm). **(D)** Quantitative assessment of TRAP-positive cells. The results represent the mean ± SD of three independent samples. *p < 0.05, **p < 0.01, ***p < 0.001 vs. Control (RANKL+, Ms-).

Red yeast rice (RYR) has been used in East Asian countries for thousands of years as a food supplement or herbal medicine and is produced by the fermentation of rice with *Monascus purpureus* (Nguyen et al., 2017). According to numerous previous researches, RYR has been reported to maintain bone health (Wong and Rabie, 2008; Wang et al., 2015; Wu et al., 2020) and be effective against a variety of diseases such as hyperlipidemia, osteoporosis and cardiovascular disease (Wang et al., 2015; Liu et al., 2017; Xiong et al., 2017; Wang et al., 2019; Wu et al., 2020). Notably, *Monascus purpureus* has been shown to produce many functional monascus pigments, applied as a food colorant, functional foods or treatment for some diseases (Patakova, 2013). Yellow pigments monascin (Ms, Figure 1A) has a potential role in alleviating metabolic syndrome (Lin et al., 2017). It has anti-inflammatory, antioxidant and other properties (Lin et al., 2017; Zhang et al., 2020). The effect of Ms on OCs has not been investigated yet.

This study explores the role of Ms in RANKL-induced OC differentiation and bone resorption *in vitro* and investigates its potential mechanism. Ms may provide a treatment strategy for osteoporosis.

## Materials and methods

### Materials

RAW264.7 cells were purchased from the Institute of Basic Medicine of Peking Union Medical College (Beijing, China). High glucose Dulbecco minimum essential medium (DMEM) and fetal bovine serum (FBS) were sourced from Gibco Company (Rockford, IL, United States). Recombinant Mouse RANKL (462-TEC-010/CF) was obtained from R&D Systems (R&D, Minneapolis, United States). Leukocyte Acid Phosphatase (TRAP) Kits (G1492), bovine bone slices (IDS, Jelling, Denmark), 1% toluidine blue O solution in sodium borate (G3663) were obtained from the Solarbio company (Beijing, China). Monascin (Ms, CAS: 21,516-68-7, purity>98% by HPLC) was purchased from Chengdu Desite Bio-Technology Company (Chengdu, China). All cell culture plasticware was supplied by Corning (NY, United States). Primary antibodies of p-p38(Thr180/Tyr182), p-ERK (Thr202/Tyr204), p-JNK(Thr183/Tyr185) and NFATc1were products of Cell Signaling Technology (Beverly, MA, United States). Primary antibodies of c-Fos and secondary antibodies were purchased from the Proteintech group (Rocky Hill, United States).

### Cell culture and viability assay

RAW264.7 cells were cultured in DMEM supplemented with 10% FBS (complete DMEM) at 37 °C in a 5% CO_2_ incubator with humidified air.

To determine the effect of Ms on the viability of RAW264.7 cells, Cell Counting Kit-8 (CCK-8) assays were used. The cells at a density of 8 × 10^3^ cells per well were seeded into a sterile 96-well plate, and incubated for 24 h to allow the cells to attach to the plate. After incubation with Ms at concentrations of 0–20 μM for 72 h, 100 μL 10% CCK-8 solution was added to replace the original medium, followed by 4 h of incubation at 37°C, and the absorbance at 450 nm was measured using a microplate reader.

### TRAP staining assay

TRAP staining was conducted to identify the morphology of mature OCs. RAW264.7 cells were cultured overnight in a 24-well plate at a density of 1×10^4^ cells per well for adherence and then stimulated with 0, 5, 10, 15 and 20 μM Ms respectively in combination with RANKL 50 ng/ml. After 5 days, the cells were fixed with 4% paraformaldehyde for 5 min at 4°C and then stained with the TRAP staining kit according to the manufacturer’s instructions. Cells with more than three nuclei were identified as TRAP-positive multinucleated cells (TRAP (+)), which were counted and photographed using a light microscope.

### Bone resorption assay

RAW264.7 cells (8 × 10^3^ cells per well) were seeded in a 96-well plate containing a sterile bovine bone slice, using the same methods of stimulation and cultivation as described previously. After a 7-days incubation period, all slices were washed in an ultrasonic instrument with 0.1% aqueous ammonia to remove the cells adhered to the surface. Subsequently, the bone slices were stained with 1% toluidine blue solution as described in the previous experiment described. Resorption pits were photographed using a microscope, and the percentage resorption was analyzed using Image-Pro Plus.

### F-actin ring formation assay

RAW264.7 cells at a density of 1 × 10^4^ cells per well were induced in 24-well plates in a complete medium with RANKL (50 ng/ml) and Ms (0, 5, 10, 15 and 20 μM) for 5 days. The cells were fixed in 4% paraformaldehyde for 20 min, permeabilized with 0.1% TritonX-100 for 10 min, and stained with rhodamine-conjugated phalloidin for 40 min at 4 C to visualize F-actin cytoskeleton. Finally, the actin rings of mature OCs were captured using a fluorescence microscope. The fluorescence images were processed by Image-Pro Plus software.

### Quantitative real-time PCR (RT-qPCR)

RAW264.7 cells (4×10^4^ cells per well) were seeded in 12-well plates using the same methods. Total RNA was extracted from cells by TRIzol reagent. After isolation, 3 μg total RNA from each sample was reverse transcribed into cDNA by Oligo (dT) and M-MLV reverse transcriptase. Thereafter, using SYBR Green detection and specific primers (listed in Table 1), the RT-generated DNA (0.5 μL) was amplified by a Bio-rad real-time PCR detection system. On completion of the reaction, gene expression values were calculated following the relative quantification method (2^−ΔΔCT^; means ± SD of triplicate determination).

**TABLE 1:** RT-qPCR related primers.

Gene Names	Forward primer sequence (5′-3′)	Reverse primer sequence (5′-3′)
*β-actin*	GGC​TGT​ATT​CCC​CTC​CAT​CG	CCA​GTT​GGT​AAC​AAT​GCC​ATG​T
*c-Fos*	CGG​GTT​TCA​ACG​CCG​ACT​A	TGG​CAC​TAG​AGA​CGG​ACA​GAT
*NFATc1*	GCC​TTT​TGC​GAG​CAG​TAT​CTG	GCT​GCA​CCT​CGA​TCC​GAA​G
*MMP-9*	GGA​CCC​GAA​GCG​GAC​ATT​G	CGT​CGT​CGA​AAT​GGG​CAT​CT
*CTSK*	CTC​GGC​GTT​TAA​TTT​GGG​AGA	TCG​AGA​GGG​AGG​TAT​TCT​GAG​T
*TRAP*	CAC​TCC​CAC​CCT​GAG​ATT​TGT	CAT​CGT​CTG​CAC​GGT​TCT​G
*ITG-β3*	CCT​CTT​CGG​CTA​CTC​GGT​C	CCA​GTC​CGG​TTG​GTA​TAG​TCA​TC
*OSCAR*	CCG​TGC​TGA​CTT​CAC​ACC​AA	GGG​GTG​ACA​AGG​CCA​CTT​TT
*DC-STAMP*	CTG​TGT​CCT​CCC​GCT​GAA​TAA	AGC​CGA​TAC​AGC​AGA​TAG​TCC

### Western blot analysis

The cells were washed three times with ice-cold PBS, and lysed in radioimmunoprecipitation assay (RIPA) buffer supplemented with Protease Inhibitor Cocktail and phenylmethylsulfonyl fluoride (PMSF) to obtain total protein. Proteins were quantitated using a bicinchoninic acid protein assay kit, separated on a 10% polyacrylamide gel, and transferred to a polyvinylidene difluoride membrane. Subsequently, the membranes were blocked with 5% skimmed milk for 1 h at room temperature, and then incubated with the indicated primary antibodies including c-Fos (1:500), NFATc1 (1:1,000), p-p38 (1:1,000), p-JNK (1:1,000), p-ERK (1:1,000) and GAPDH (1:1,000) at 4 °C overnight. Then, the membranes were incubated with the corresponding horseradish peroxidase-conjugated secondary antibodies for 2 h, visualized with the ECL system, and analyzed by ImageJ software.

### Statistical analysis

Statistical analysis was performed with SPSS 25.0 software. The data are expressed as mean ± standard deviation (SD). Two-group comparisons were conducted using the Students’ *t*-test, multiple-group comparisons were conducted using one-way ANOVA, and any two-group comparisons were conducted by SNK-q test. Differences were considered to be statistically significant if *p* < 0.05.

## Results

### Ms inhibited RANKL-induced osteoclastogenesis

Ms treatment at concentrations of 0–20 μM for 72 h did not affect the viability of OC precursors (Figure 1B). TRAP is considered a histochemical marker of OCs(Lv et al., 2015). A TRAP staining assay was used to explore whether Ms suppressed RANKL-induced OC formation. As shown in Figures 1C,D, the number of TRAP-positive multinuclear cells increased substantially in response to RANKL stimulation. Still, Ms treatment suppressed the purple giant multinucleated cells induced by RANKL in a dose-dependent manner. Collectively, these results indicate that Ms inhibits the OC formation and differentiation of RANKL-stimulated RAW264.7 cells.

### Ms affected OC morphology and bone resorption *in vitro*

**FIGURE 2:**
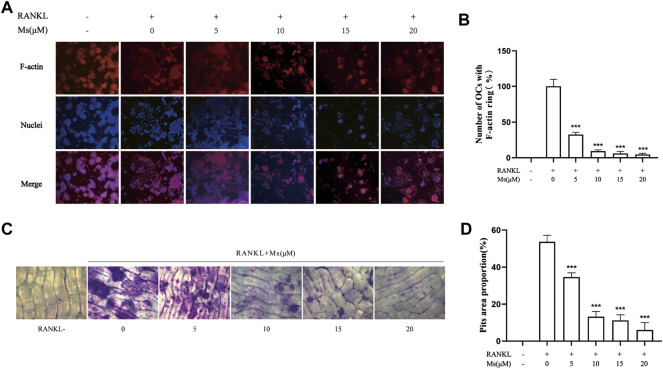
Ms inhibits formation of the F-actin ring and osteoclast resorptive function induced by RANKL *in vitro*. RAW264.7 macrophages were cultured in 24-well plates with or without Ms (5, 10, 15 and 20 μM) in the presence of 50 ng/ml RANKL for 5 days. **(A)** Representative images of F-actin rings; F-actin: red, nuclei: blue, original magnification ×200 (Scale bar = 200 μm) **(B)** Quantitative analyses of osteoclast numbers with intact F-actin ring. RAW264.7 macrophages were cultured on a bone slice in 96-well plates with or without Ms (5, 10, 15 and 20 μM) in the presence of 50 ng/ml RANKL for 7 days. **(C)** Resorption pits of osteoclasts were captured by a light microscope. Original magnification ×200 (Scale bar = 200 μm) **(D)** The resorbed areas were calculated using Image **(J)**. The results represent the mean ± SD of three independent samples. *p < 0.05, **p < 0.01, ***p < 0.001 vs. Control (RANKL+, Ms-).

Mature OCs play an essential role in polarization and adherence in bone resorption due to their unique morphological characteristics, consisting of an actin ring structure and a ruffled border membrane (Garbe et al., 2012; Georgess et al., 2014). In this study, F-actin rings were visualized by phalloidin staining and bone resorption pits were identified by toluidine blue staining. As illustrated in Figures 2A–D, numerous F-actin rings and bone resorption pits were generated in RANKL-induced RAW264.7-OCs, which were significantly reduced following Ms treatment in a dose-dependent manner. These results indicate that Ms inhibits the OC bone resorption.

### Ms reduced the expression of OC-related genes

**FIGURE 3:**
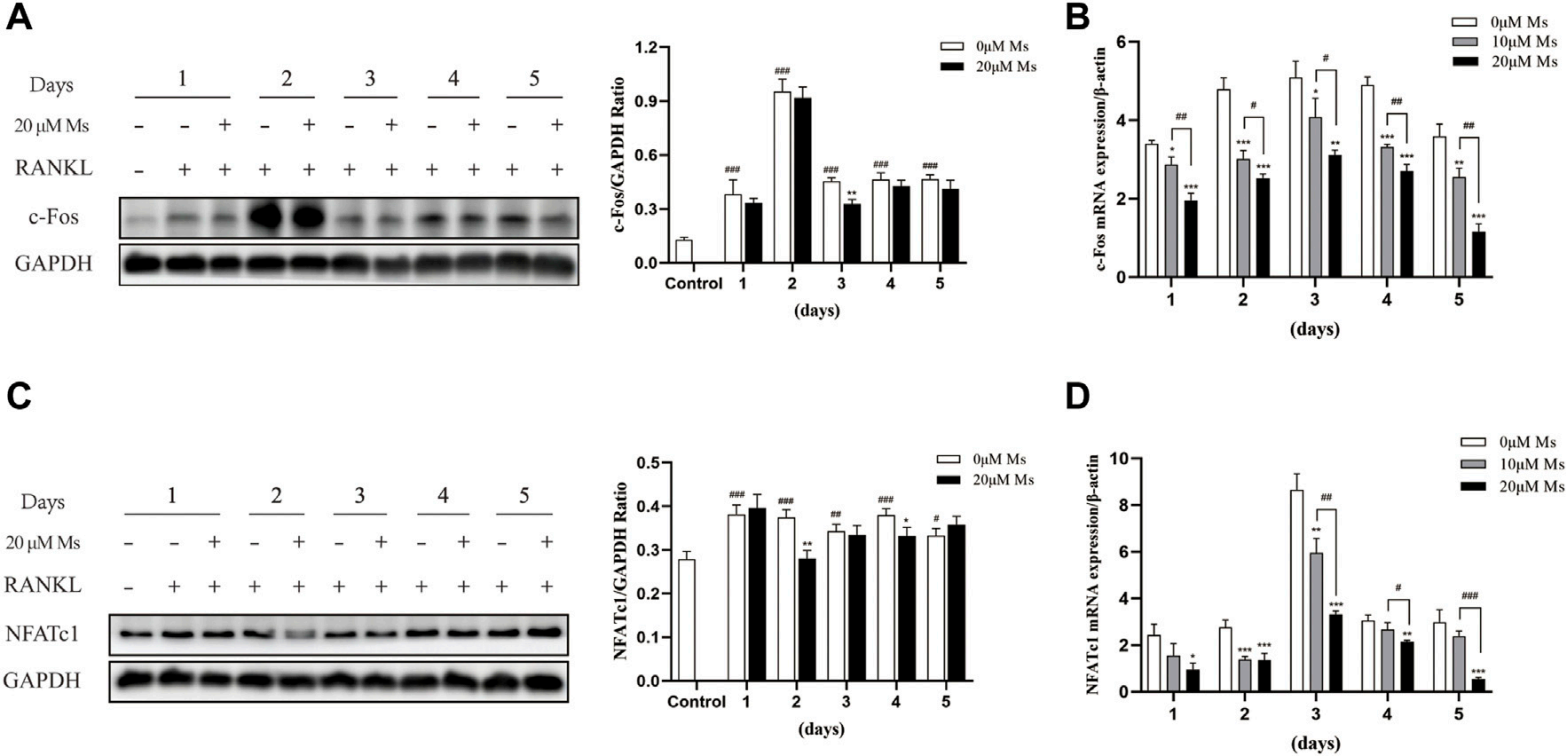
Effect of Ms on NFATc1 and c-Fos expression in RANKL-induced RAW264.7 cells. RAW264.7 cells were treated with varying concentrations of Ms with RANKL (50 ng/ml) for 5 days. The proteins were extracted to analyze c-Fos and NFATc1 expression by Western blot per day (A and C). The mRNA expression levels of c-Fos and NFATc1 genes were analyzed using RT-qPCR per day (B and D). Each point represents the mean ± SD. The experiments were repeated three times. #*p* < 0.05, ##*p* < 0.01 and ###*p* < 0.001 vs. Control group (RANKL-, Ms-); *p < 0.05, **p < 0.01 vs. ***p < 0.001 vs. corresponding time 0 μM Ms group (RANKL+, Ms-).

**FIGURE 4:**
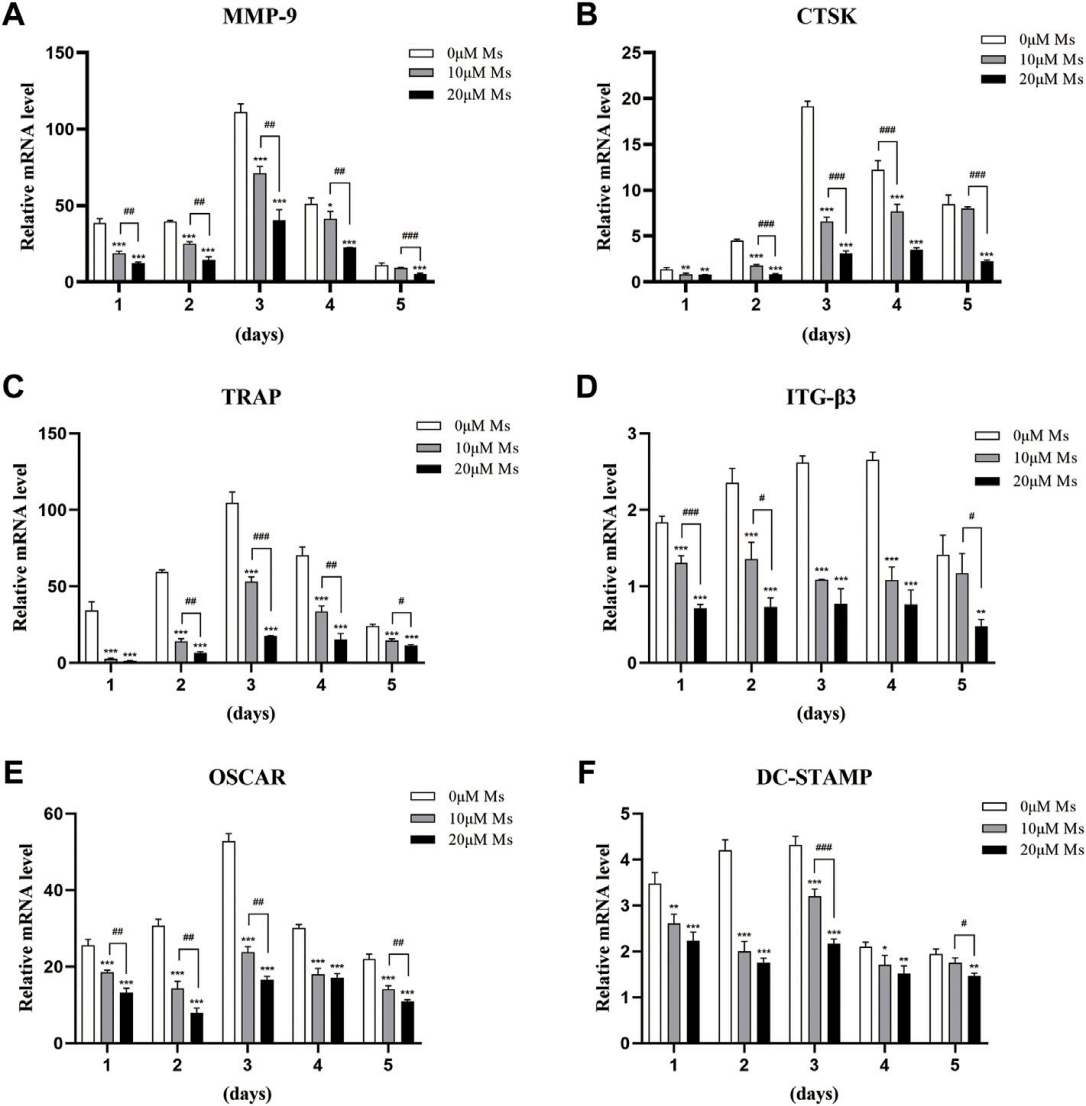
Ms inhibits the RANKL-induced expression of osteoclastic marker genes. RAW264.7 cells were treated with or without Ms (10 and 20 μM) in the presence of RANKL (50 ng/ml) after a 1–5 days culture period. The mRNA expression levels of osteoclast-specific genes, such as MMP-9 **(A)**, CTSK **(B)**, TRAP **(C)**, ITG-β3 **(D)**, OSCAR **(E)**, and DC-STAMP **(F)**, were analyzed using RT-qPCR. *p < 0.05, **p < 0.01 vs. ***p < 0.001 vs. corresponding time 0 μM Ms group (RANKL+, Ms-); #*p* < 0.05, ##*p* < 0.01 and ###*p* < 0.001 vs. corresponding time 10 μM Ms group (RANKL+, Ms+).

Western blot analysis was carried out to investigate whether Ms inhibited c-Fos and NFATc1. As shown in Figures 3A,C, Ms significantly inhibited c-Fos and NFATc1 protein levels. Furthermore, RT-qPCR was performed to analyze expression levels of OC-specific genes in the absence or presence of Ms. As shown in Figures 3B,D, Ms had a strong inhibitory effect on c-Fos and NFATc1 genes, essential transcription factors and master regulators of osteoclastogenesis. In addition, we found that several NFATc1-regulated genes, including MMP-9, CTSK, TRAP, ITG-β3, OSCAR and DC-STAMP, were upregulated in RANKL-induced RAW264.7-OCs, but downregulated by Ms in a dose-dependent manner (Figure 4). These results suggest that Ms inhibits osteoclastogenesis via suppressing the expression of these genes.

### Ms suppressed the activation of the MAPKs pathways

**FIGURE 5:**
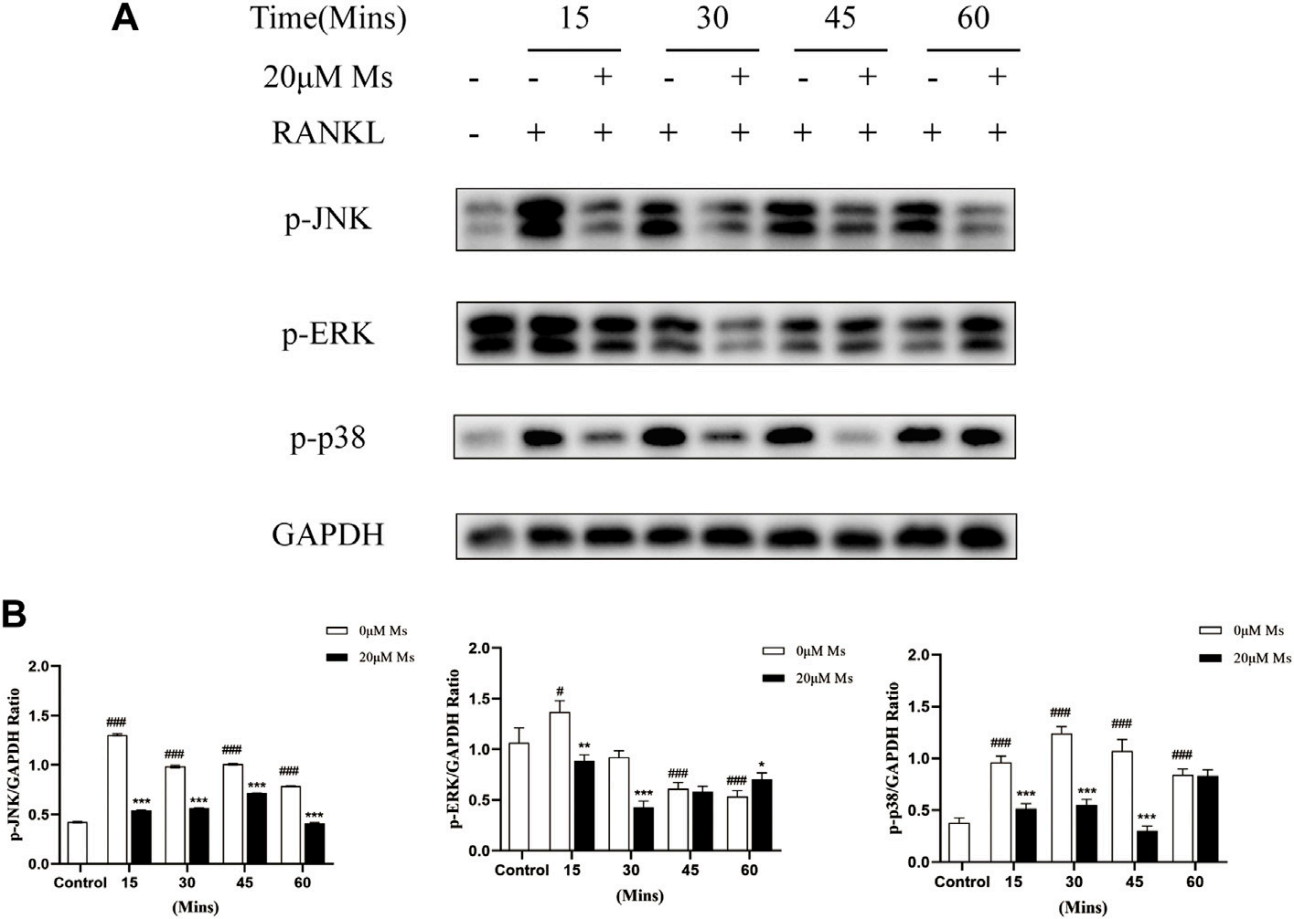
Ms inhibits RANKL-induced osteoclast differentiation by suppressing MAPKs signaling pathways. RAW264.7 cells were treated with or without Ms (20 μM) in the presence of RANKL. Protein levels of MAPKs activation, including JNK, ERK and p38, were detected by western blot at 0, 15, 30, 45 and 60 min. The results were expressed as means ± SD from three independent experiments. #*p* < 0.05, ##*p* < 0.01 and ###*p* < 0.001 vs. Control group (RANKL-, Ms-); *p < 0.05, **p < 0.01 vs. ***p < 0.001 vs. corresponding time 0 μM Ms group (RANKL+, Ms-).

RANKL bind to its receptor RANK to activate a series of intracellular signaling pathways involved in OC differentiation, proliferation and survival (Leibbrandt and Penninger, 2009), including MAPKs pathways, which activate various transcription factors through phosphorylation (Ono and Nakashima, 2018). Firstly, western blot analysis was carried out to investigate whether Ms inhibited the activation and phosphorylation of MAPKs. Treatment with RANKL significantly increased the phosphorylation of JNK and p38 within 60 min, except ERK (Figure 5). Treatment with Ms for 15 min attenuated the phosphorylation of p38, JNK and ERK (Figure 5). These findings suggest that Ms inhibits RANKL induced osteoclastogenesis and bone resorption by suppressing MAPKs signal pathways in OCs.

## Discussion

Osteoclasts are essential in the complex process of bone remodeling and are related to lytic bone disorders, including osteoporosis and bone tumor metastasis, according to previous studies (Novack and Teitelbaum, 2008). Inhibiting OC formation is a promising strategy for these diseases. However, synthetic drugs usually cause severe side effects relative to natural components from herbal medicine. In this study, we performed a TRAP staining assay and observed that Ms inhibited RANKL-induced osteoclastogenesis. In addition, bone resorption was suppressed by Ms *in vitro*. Ms also suppressed OCs-typical genes in a dose-dependent manner. Furthermore, Ms inhibited the RANKL-induced phosphorylation of p38, JNK and ERK, and activation of c-Fos and NFATc1 in RANKL-induced RAW264.7-OCs.

The RAW264.7 cell linage is well-accepted and vastly used as a cellular model in osteoclastogenic research, supporting the exploration of the biological mechanisms of osteoclastogenesis (Collin-Osdoby and Osdoby, 2012). Mature OCs are giant, multinuclear, and polarized cells characterized by rearrangements of the actin cytoskeleton and a sealed compartment between the bone surface and basal membrane. Mature OCs are responsible for the digestion of the bone matrix (Boyle et al., 2003). In the first stage of this study, we defined TRAP-positive OCs and bone resorption area as indicators. The results revealed that Ms treatment significantly decreased the number of TRAP (+) cells, bone resorption area and F-actin rings compared to the RANKL-induced RAW264.7-OCs group. The findings suggest that Ms inhibits RANKL-mediated osteoclastogenesis and bone resorption. The underlying mechanisms of the inhibitory properties of Ms were investigated in subsequent experiments.

NFATc1 is a common target gene of both NF-κB and AP-1 in the early phase of RANKL-stimulated osteoclastogenesis. The former is recruited by the NFATc1 promoter, and the latter, containing c-Fos, assists NFATc1 (Ishida et al., 2002; Asagiri and Takayanagi, 2007). Cumulative evidence also indicates that c-Fos activation is a prerequisite for NFATc1 because RANKL-induced NFATc1 is completely abrogated in c-Fos-deficient cells (Takayanagi et al., 2002; Matsuo et al., 2004). In the second stage of our study, the essential transcription factors of c-Fos and NFATc1 were measured. Subsequently, we detected the OC-specific gene expression regulated by NFATc1, including TRAP, CTSK, MMP-9, OSCAR, DC-STAMP, and ITG-β3. Ms suppressed the activation of c-Fos and NFATc1 and reduced the expression of OC formation/function-related genes in a dose-dependent manner.

The stimuli of extracellular signaling transmission to coordinated intracellular molecules by MAPKs activation has been demonstrated to be significant for osteoclastogenesis (Lee et al., 2018; Qu et al., 2021). Both M-CSF and RANKL act through MAPKs, including ERK, JNK, and p38 signaling as cultured bone marrow/RAW264.7 cells differentiate into OCs (Ross, 2006; Lee et al., 2016). In the third stage of our study, we explored the inhibitory effect of Ms on the MAPKs signaling pathway. A series of experiments indicated that Ms attenuated the phosphorylation of p38, JNK and ERK. These results suggest that Ms inhibited RANKL-induced RAW264.7-OCs via blocking the MAPKs signaling pathways.

**FIGURE 6:**
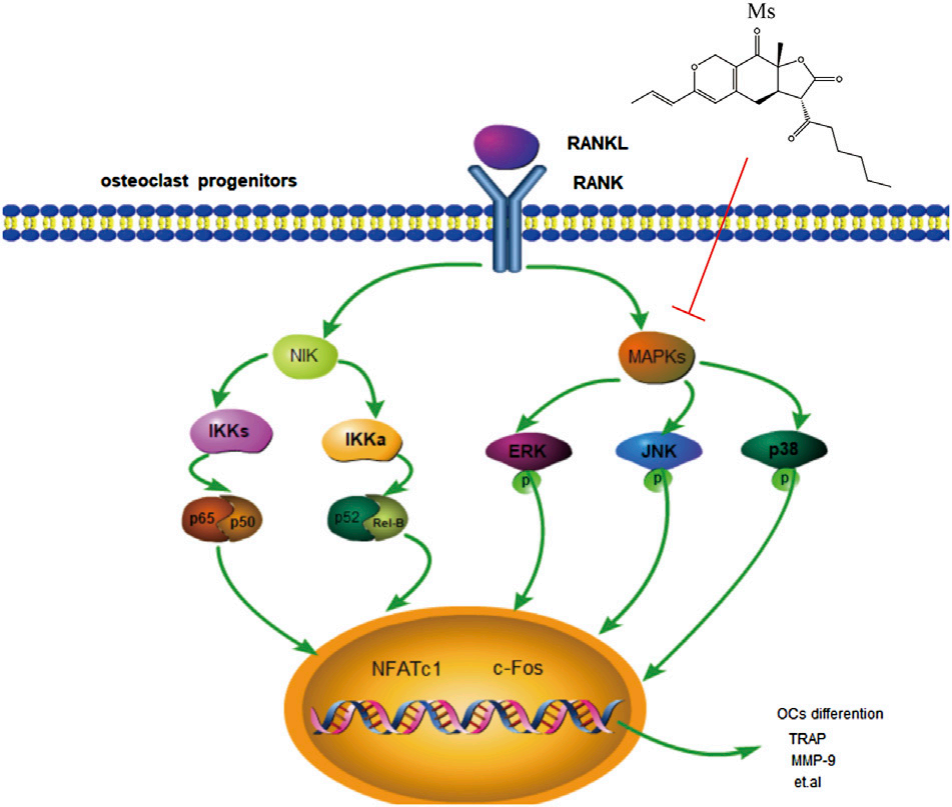
Schematic representation of the mechanism by which Ms inhibits osteoclastogenesis in RAW264.7 cells. RANKL induces MAPKs activation, including activation of JNK, p38 and ERK. Subsequently, the expression of osteoclast-typical genes, MMP-9, CTSK, TRAP, ITG-β3, OSCAR, and DC-STAMP are upregulated. Our results show that Ms inhibits osteoclastogenesis by inhibiting MAPKs activation. The sign (↓) means promoting. The sign (┴) means inhibiting.

Taken together, this study was shown that Ms has potential anti-osteoclastogenic effects by inhibiting the MAPKs signaling pathways activation (Figure 6). Furthermore, Ms inhibits the transcription factors NFATc1 and c-Fos, resulting in decreased expression of OC-specific genes such as TRAP, CTSK, MMP-9, OSCAR, DC-STAMP, and ITG-β3. This study provides fundamental knowledge about the effects of Ms on osteoclastogenesis and insight into the therapeutic potential for diseases such as osteoporosis. In the future, animal studies will be performed to determine whether Ms retains its anti-osteoclastogenic effects and its impact on the MAPKs pathways *in vivo*.

## Data Availability

The raw data supporting the conclusions of this article will be made available by the authors, without undue reservation.
